# {3-Methyl-2-[(1-oxido-2-naphth­yl)methyl­idene­amino-κ^2^ *O*,*N*]butano­ato-κ*O*}(1*H*-pyrazole-κ*N* ^2^)nickel(II)

**DOI:** 10.1107/S1600536810032472

**Published:** 2010-08-18

**Authors:** Qin-Long Peng, Gan-Qing Zhao, Li-Hua Chen, Ling-Wei Xue

**Affiliations:** a School of Chemistry and Chemical Engineering, Pingdingshan University, Pingdingshan 467000, People’s Republic of China

## Abstract

In either of the two independent mol­ecules within the asymmetric unit of the title compound, [Ni(C_16_H_15_NO_3_)(C_3_H_4_N_2_)], the Ni^II^ atom is coordinated by the two N atoms and two O atoms in a distorted square-planar geometry. The crystal packing is stabilized by strong and weak inter­molecular C—H⋯O hydrogen bonds, as well as weak centroid–centroid π-stacking inter­actions [centroid–centroid separation = 3.526 (3) Å].

## Related literature

For complexes of Schiff base ligands composed of salicyl­aldehyde, 2-formyl­pyridine or their analogues, see: Li *et al.* (2010 ▶); Vergopoulos *et al.* (1993 ▶); Usman *et al.* (2003 ▶). For related structures, see: Basu Baul *et al.* (2007 ▶); Ebel & Rehder (2003 ▶); Maniukiewicz & Bukowska-Strzyżewska (2001 ▶); Xue *et al.* (2009 ▶); Qiu *et al.* (2008 ▶). For the synthesis, see: Plesch *et al.* (1997 ▶).
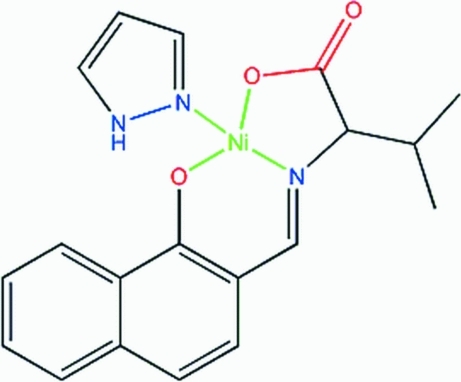


## Experimental

### 

#### Crystal data

[Ni(C_16_H_15_NO_3_)(C_3_H_4_N_2_)]
*M* *_r_* = 396.08
Orthorhombic, 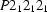

*a* = 11.5089 (11) Å
*b* = 16.6194 (16) Å
*c* = 18.9934 (19) Å
*V* = 3632.9 (6) Å^3^
*Z* = 8
Mo *K*α radiation
μ = 1.09 mm^−1^
*T* = 296 K
0.30 × 0.30 × 0.25 mm

#### Data collection

Bruker SMART APEX CCD area-detector diffractometer
Absorption correction: multi-scan (*SADABS*; Sheldrick, 2003 ▶) *T*_min_ = 0.735, *T*_max_ = 0.772
18967 measured reflections
6400 independent reflections
4910 reflections with *I* > 2σ(*I*)
*R*_int_ = 0.045

#### Refinement

*R*[*F* ^2^ > 2σ(*F* ^2^)] = 0.044
*wR*(*F* ^2^) = 0.108
*S* = 1.02
6400 reflections
473 parameters
H-atom parameters constrained
Δρ_max_ = 0.53 e Å^−3^
Δρ_min_ = −0.36 e Å^−3^
Absolute structure: Flack (1983 ▶), 2792 Friedel pairs
Flack parameter: −0.015 (16)

### 

Data collection: *SMART* (Bruker, 2000 ▶); cell refinement: *SAINT* (Bruker, 2000 ▶); data reduction: *SAINT*; program(s) used to solve structure: *SHELXS97* (Sheldrick, 2008 ▶); program(s) used to refine structure: *SHELXL97* (Sheldrick, 2008 ▶); molecular graphics: *SHELXTL* (Sheldrick, 2008 ▶); software used to prepare material for publication: *SHELXTL*.

## Supplementary Material

Crystal structure: contains datablocks global, I. DOI: 10.1107/S1600536810032472/jj2044sup1.cif

Structure factors: contains datablocks I. DOI: 10.1107/S1600536810032472/jj2044Isup2.hkl

Additional supplementary materials:  crystallographic information; 3D view; checkCIF report

## Figures and Tables

**Table:** 

Ni1—O1	1.805 (3)
Ni1—N1	1.833 (3)
Ni1—O2	1.852 (3)
Ni1—N2	1.900 (3)

**Table:** 

O1—Ni1—N1	94.55 (15)
O1—Ni1—O2	176.92 (16)
N1—Ni1—O2	86.20 (15)
O1—Ni1—N2	89.98 (16)
N1—Ni1—N2	173.95 (16)
O2—Ni1—N2	89.49 (16)

**Table 2:** Hydrogen-bond geometry (Å, °)

*D*—H⋯*A*	*D*—H	H⋯*A*	*D*⋯*A*	*D*—H⋯*A*
C7—H7⋯O2^i^	0.93	2.59	3.476 (6)	159
C14—H14⋯O6^ii^	0.98	2.42	3.318 (6)	153
C18—H18⋯O6^iii^	0.93	1.87	2.798 (5)	178
C37—H37⋯O3^iv^	0.93	1.85	2.756 (5)	163

Symmetry codes: (i) 
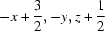
; (ii) 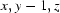; (iii) 
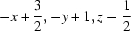
; (iv) 
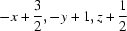
.

**Table 3:** Weak *Cg*–*Cg* inter­molecular inter­actions of (I) (Å)

Distance	*Cg*1–*Cg*10	*Cg*3–*Cg*9	*Cg*4–*Cg*7	*Cg*4–*Cg*9
Centroid–centroid distance	3.940 (3)	3.709 (2)	3.526 (3)	3.932 (3)

Notes: *CgI*–*CgJ* = centroid–centroid distance between planes *I* and *J* (Å); *Cg*1: Ni1/O2/C13/C12/N1; *Cg*3: Ni1/O1/C1/C10/C11/N1; *Cg*4: C1/C2/C3/C4/C9/C10; *Cg*7: Ni2/O5/C32/C31/N4; *Cg*9: Ni2/O4/C20/C29/C30/N4; *Cg*10: C20/C21/C22/C23/C28/C29.

**Fig. 1.**
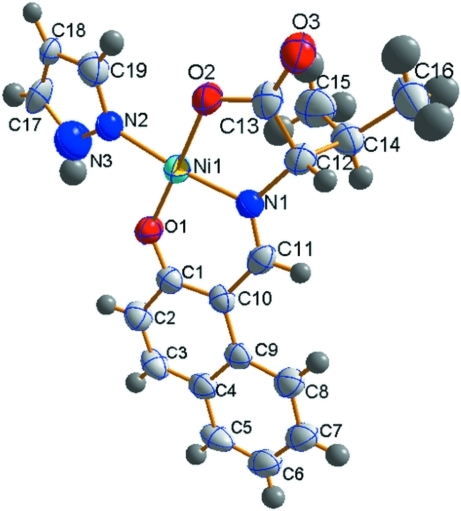
The structure of the title compound, (I), showing 30% probability displacement ellipsoids and the atom-numbering scheme.

**Fig. 2.**
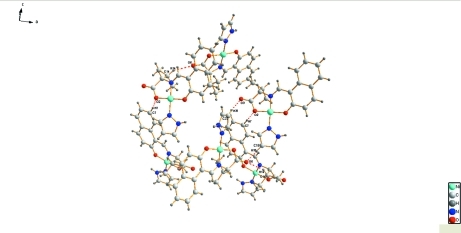
A view of the crystal packing. Hydrogen bonds are shown as red dashed lines.

## supplementary crystallographic information

### Comment

Complexes of Shiff base ligands composed of salicylaldehyde, 2-formylpyidine or their analogues, and α-amino acid always have attracted attention due to the important biomolecules–α-amino acid and manifold structure (Vergopoulos *et al.*, 1993; Usman *et al.*, 2003; Li *et al.*, 2010). Several structural studies have been performed on Shiff base transition metal complex derived from 1-hydroxy-2-naphthaldehyde and α-amino acid (Ebel *et al.*, 2003; Qiu *et al.*, 2008; Xue *et al.*, 2009). We report here the crystal structure of the title Ni^II^complex, (I), [Ni(C_16_H_15_N_1_O_3_)(C_3_H_4_N_2_)].

In the title complex, the Ni^II^atom is in a distorted square-planar coordination geometry (Fig. 1; table 1). Three basal positions are occupied by three donor atoms from the tridentate Schiff base ligand, which furnishes an O–N–O donor set, with the fourth position occupied by one N atom from the pyrazole ligand.

The dihedral angle between the mean planes of the naphthalene and pyrazole rings is 16.(7)°. Strong and weak intermolecular C—H···O hydrogen bonds (Fig. 2; Table 2) and weak *Cg*···*Cg*π-stacking interactions [shortest centroid-centroid separation = 3.526 (3) Å] contribute to crystal packing (Table 3).

### Experimental

The title compound was synthesized as described in the literature (Plesch *et al.*, 1997). To L-valine (1.00 mmol) and potassium hydroxide (1.00 mmol) in 10 ml of methanol and 5 ml of water was added 2-Hydroxy-1-naphthaldehyde (1.00 mmol in 10 ml of methanol) dropwise.The yellow solution was stirred for 2.0 h at 333 K. The resultant mixture was added dropwise to Ni (II) nitrate Hexahydrate (1.00 mmol) and pyrazole (1.00 mmol) in an aqueous methanolic solution (20 ml, 1:1 *v*/*v*), and heated with stirring for 4.0 h at 333 K. The brown solution was filtered and left for several days, brown crystals had formed that were filtered off, washed with water, and dried under vacuum.

### Refinement

In (I), All H atoms were positioned geometrically and refined as riding atoms, with C—H = 0.93 Å (CH), with C—H = 0.96 Å (CH_3_) and *U*_iso_(H) = 1.5*U*_eq_(C), and with N—H = 0.86 Å (NH) and *U*_iso_(H) = 1.2*U*_eq_(N).

### Crystal data

**Table:**

[Ni(C_16_H_15_NO_3_)(C_3_H_4_N_2_)]	*F*(000) = 1648
*M**_r_* = 396.08	*D*_x_ = 1.448 Mg m^−^^3^
Orthorhombic, *P*2_1_2_1_2_1_	Mo *K*α radiation, λ = 0.71073 Å
Hall symbol: P 2ac 2ab	Cell parameters from 4302 reflections
*a* = 11.5089 (11) Å	θ = 2.4–21.1°
*b* = 16.6194 (16) Å	µ = 1.09 mm^−^^1^
*c* = 18.9934 (19) Å	*T* = 296 K
*V* = 3632.9 (6) Å^3^	Block, brown
*Z* = 8	0.30 × 0.30 × 0.25 mm

### Data collection

**Table:**

Bruker SMART APEX CCD area-detector diffractometer	6400 independent reflections
Radiation source: fine-focus sealed tube	4910 reflections with *I* > 2σ(*I*)
graphite	*R*_int_ = 0.045
φ and ω scans	θ_max_ = 25.1°, θ_min_ = 2.1°
Absorption correction: multi-scan (*SADABS*; Sheldrick, 2003)	*h* = −11→13
*T*_min_ = 0.735, *T*_max_ = 0.772	*k* = −19→19
18967 measured reflections	*l* = −22→22

### Refinement

**Table:**

Refinement on *F*^2^	Secondary atom site location: difference Fourier map
Least-squares matrix: full	Hydrogen site location: inferred from neighbouring sites
*R*[*F*^2^ > 2σ(*F*^2^)] = 0.044	H-atom parameters constrained
*wR*(*F*^2^) = 0.108	*w* = 1/[σ^2^(*F*_o_^2^) + (0.0469*P*)^2^ + 0.9539*P*] where *P* = (*F*_o_^2^ + 2*F*_c_^2^)/3
*S* = 1.02	(Δ/σ)_max_ < 0.001
6400 reflections	Δρ_max_ = 0.53 e Å^−^^3^
473 parameters	Δρ_min_ = −0.36 e Å^−^^3^
0 restraints	Absolute structure: Flack (1983), 2792 Friedel pairs [PLEASE CHECK]
Primary atom site location: structure-invariant direct methods	Flack parameter: −0.015 (16)

### Special details

**Table:**

Geometry. All e.s.d.'s (except the e.s.d. in the dihedral angle between two l.s. planes) are estimated using the full covariance matrix. The cell e.s.d.'s are taken into account individually in the estimation of e.s.d.'s in distances, angles and torsion angles; correlations between e.s.d.'s in cell parameters are only used when they are defined by crystal symmetry. An approximate (isotropic) treatment of cell e.s.d.'s is used for estimating e.s.d.'s involving l.s. planes.
Refinement. Refinement of *F*^2^ against ALL reflections. The weighted *R*-factor *wR* and goodness of fit *S* are based on *F*^2^, conventional *R*-factors *R* are based on *F*, with *F* set to zero for negative *F*^2^. The threshold expression of *F*^2^ > σ(*F*^2^) is used only for calculating *R*-factors(gt) *etc*. and is not relevant to the choice of reflections for refinement. *R*-factors based on *F*^2^ are statistically about twice as large as those based on *F*, and *R*- factors based on ALL data will be even larger.

### Fractional atomic coordinates and isotropic or equivalent isotropic displacement parameters (Å^2^)

**Table:**

	*x*	*y*	*z*	*U*_iso_*/*U*_eq_	
Ni1	0.75779 (5)	0.07571 (3)	0.05529 (3)	0.04451 (17)	
Ni2	0.29080 (5)	0.80826 (3)	0.31116 (3)	0.04473 (17)	
C1	0.5662 (4)	0.1225 (3)	0.1366 (3)	0.0445 (12)	
C2	0.4616 (4)	0.1673 (3)	0.1395 (3)	0.0560 (13)	
H2	0.4354	0.1933	0.0992	0.067*	
C3	0.3991 (5)	0.1734 (3)	0.1993 (3)	0.0598 (14)	
H3	0.3285	0.2005	0.1986	0.072*	
C4	0.4389 (5)	0.1392 (3)	0.2630 (3)	0.0532 (14)	
C5	0.3769 (5)	0.1514 (3)	0.3268 (3)	0.0623 (15)	
H5	0.3064	0.1787	0.3260	0.075*	
C6	0.4201 (6)	0.1231 (3)	0.3901 (3)	0.0672 (16)	
H6	0.3792	0.1317	0.4316	0.081*	
C7	0.5240 (5)	0.0821 (3)	0.3912 (3)	0.0588 (14)	
H7	0.5536	0.0633	0.4337	0.071*	
C8	0.5844 (5)	0.0688 (3)	0.3298 (2)	0.0531 (13)	
H8	0.6537	0.0401	0.3318	0.064*	
C9	0.5449 (4)	0.0969 (3)	0.2639 (3)	0.0432 (12)	
C10	0.6076 (4)	0.0857 (3)	0.1986 (2)	0.0402 (10)	
C11	0.7082 (4)	0.0369 (2)	0.1959 (2)	0.0435 (11)	
H11	0.7295	0.0102	0.2369	0.052*	
C12	0.8739 (4)	−0.0294 (3)	0.1441 (2)	0.0464 (12)	
H12	0.9156	−0.0200	0.1883	0.056*	
C13	0.9522 (5)	−0.0079 (3)	0.0842 (3)	0.0482 (12)	
C14	0.8356 (5)	−0.1181 (3)	0.1417 (3)	0.0567 (14)	
H14	0.7791	−0.1253	0.1798	0.068*	
C15	0.7731 (6)	−0.1393 (3)	0.0738 (3)	0.0812 (18)	
H15A	0.8284	−0.1428	0.0361	0.122*	
H15B	0.7170	−0.0983	0.0632	0.122*	
H15C	0.7344	−0.1900	0.0791	0.122*	
C16	0.9363 (6)	−0.1746 (4)	0.1576 (4)	0.092 (2)	
H16A	0.9917	−0.1723	0.1199	0.139*	
H16B	0.9077	−0.2286	0.1622	0.139*	
H16C	0.9731	−0.1585	0.2007	0.139*	
N3	0.6506 (5)	0.1453 (3)	−0.0732 (3)	0.0952 (18)	
H3A	0.5839	0.1544	−0.0544	0.114*	
C18	0.7893 (4)	0.1369 (2)	−0.1493 (2)	0.0391 (10)	
H18	0.8320	0.1392	−0.1909	0.047*	
C19	0.8288 (5)	0.1139 (3)	−0.0877 (3)	0.0563 (14)	
H19	0.9049	0.0974	−0.0799	0.068*	
C20	0.1022 (4)	0.7048 (3)	0.2868 (3)	0.0452 (12)	
C21	0.0003 (5)	0.6677 (3)	0.3148 (3)	0.0568 (13)	
H21	−0.0242	0.6806	0.3601	0.068*	
C22	−0.0608 (5)	0.6140 (3)	0.2766 (3)	0.0648 (16)	
H22	−0.1264	0.5905	0.2964	0.078*	
C23	−0.0286 (5)	0.5921 (3)	0.2073 (3)	0.0541 (14)	
C24	−0.0923 (5)	0.5350 (3)	0.1676 (4)	0.0743 (18)	
H24	−0.1584	0.5120	0.1874	0.089*	
C25	−0.0604 (6)	0.5130 (4)	0.1024 (4)	0.086 (2)	
H25	−0.1032	0.4748	0.0777	0.103*	
C26	0.0366 (6)	0.5477 (4)	0.0724 (4)	0.0798 (19)	
H26	0.0583	0.5334	0.0269	0.096*	
C27	0.1015 (5)	0.6029 (3)	0.1087 (3)	0.0609 (15)	
H27	0.1663	0.6253	0.0871	0.073*	
C28	0.0734 (4)	0.6266 (3)	0.1773 (3)	0.0484 (12)	
C29	0.1375 (4)	0.6856 (3)	0.2179 (2)	0.0426 (11)	
C30	0.2362 (4)	0.7240 (2)	0.1880 (2)	0.0407 (10)	
H30	0.2559	0.7099	0.1422	0.049*	
C31	0.4015 (4)	0.8117 (3)	0.1815 (2)	0.0440 (11)	
H31	0.4416	0.7687	0.1558	0.053*	
C32	0.4824 (5)	0.8441 (3)	0.2381 (2)	0.0444 (11)	
C33	0.3714 (5)	0.8798 (3)	0.1293 (3)	0.0627 (16)	
H33	0.4463	0.9012	0.1134	0.075*	
C34	0.3129 (8)	0.8482 (5)	0.0640 (3)	0.119 (3)	
H34A	0.2402	0.8238	0.0766	0.179*	
H34B	0.3620	0.8089	0.0420	0.179*	
H34C	0.2993	0.8918	0.0319	0.179*	
C35	0.3109 (6)	0.9496 (3)	0.1654 (3)	0.086 (2)	
H35A	0.2957	0.9913	0.1317	0.130*	
H35B	0.3597	0.9703	0.2022	0.130*	
H35C	0.2388	0.9313	0.1853	0.130*	
N6	0.2387 (6)	0.8183 (3)	0.4640 (3)	0.1060 (19)	
H6A	0.1960	0.7759	0.4666	0.127*	
C37	0.3311 (4)	0.9271 (3)	0.4934 (2)	0.0412 (11)	
H37	0.3609	0.9704	0.5186	0.049*	
C38	0.3433 (4)	0.9138 (3)	0.4260 (3)	0.0578 (14)	
H38	0.3861	0.9471	0.3964	0.069*	
N1	0.7731 (3)	0.0262 (2)	0.14102 (18)	0.0418 (9)	
N2	0.7483 (4)	0.1169 (2)	−0.03787 (18)	0.0473 (9)	
C17	0.6775 (6)	0.1564 (3)	−0.1422 (3)	0.0676 (17)	
H17	0.6277	0.1741	−0.1775	0.081*	
N4	0.3010 (3)	0.7767 (2)	0.21896 (18)	0.0425 (9)	
N5	0.2891 (4)	0.8490 (2)	0.40427 (19)	0.0480 (10)	
C36	0.2692 (7)	0.8680 (4)	0.5186 (3)	0.086 (2)	
H36	0.2492	0.8608	0.5656	0.103*	
O1	0.6178 (3)	0.1191 (2)	0.07517 (16)	0.0529 (9)	
O2	0.9049 (3)	0.03658 (19)	0.03564 (16)	0.0501 (8)	
O3	1.0518 (3)	−0.0330 (2)	0.07990 (19)	0.0621 (10)	
O4	0.1546 (3)	0.7567 (2)	0.32823 (16)	0.0541 (9)	
O5	0.4362 (3)	0.8548 (2)	0.29918 (17)	0.0534 (9)	
O6	0.5840 (3)	0.8613 (2)	0.22488 (17)	0.0587 (9)	

### Atomic displacement parameters (Å^2^)

**Table:**

	*U*^11^	*U*^22^	*U*^33^	*U*^12^	*U*^13^	*U*^23^
Ni1	0.0446 (4)	0.0483 (3)	0.0407 (3)	0.0047 (3)	0.0006 (3)	0.0060 (3)
Ni2	0.0416 (4)	0.0469 (3)	0.0456 (3)	−0.0031 (3)	0.0020 (3)	−0.0033 (3)
C1	0.042 (3)	0.041 (3)	0.050 (3)	0.001 (2)	0.003 (2)	−0.002 (2)
C2	0.047 (3)	0.059 (3)	0.062 (3)	0.008 (3)	−0.003 (3)	0.004 (3)
C3	0.046 (3)	0.053 (3)	0.081 (4)	0.012 (3)	0.006 (3)	0.001 (3)
C4	0.057 (4)	0.041 (3)	0.061 (3)	−0.003 (3)	0.012 (3)	−0.004 (3)
C5	0.054 (3)	0.054 (3)	0.079 (4)	0.002 (3)	0.022 (3)	−0.013 (3)
C6	0.085 (5)	0.058 (3)	0.059 (4)	−0.008 (3)	0.020 (3)	−0.005 (3)
C7	0.070 (4)	0.059 (3)	0.048 (3)	−0.006 (3)	0.011 (3)	0.000 (3)
C8	0.058 (3)	0.051 (3)	0.051 (3)	−0.003 (3)	0.004 (3)	0.001 (2)
C9	0.046 (3)	0.033 (2)	0.050 (3)	−0.004 (2)	0.005 (2)	−0.002 (2)
C10	0.033 (2)	0.039 (2)	0.048 (3)	0.001 (2)	0.000 (2)	0.003 (2)
C11	0.047 (3)	0.046 (2)	0.038 (2)	−0.007 (2)	−0.004 (2)	0.001 (2)
C12	0.038 (3)	0.055 (3)	0.047 (3)	0.003 (2)	0.004 (2)	0.008 (2)
C13	0.045 (3)	0.048 (3)	0.051 (3)	0.001 (3)	−0.005 (2)	0.008 (2)
C14	0.045 (3)	0.051 (3)	0.074 (4)	0.005 (3)	0.018 (3)	0.013 (3)
C15	0.084 (5)	0.066 (3)	0.094 (4)	−0.007 (4)	0.009 (4)	−0.008 (3)
C16	0.073 (4)	0.072 (4)	0.132 (6)	0.027 (4)	0.030 (4)	0.038 (4)
N3	0.086 (4)	0.104 (4)	0.096 (4)	0.024 (3)	−0.001 (3)	0.011 (3)
C18	0.043 (3)	0.045 (2)	0.030 (2)	0.007 (2)	0.004 (2)	0.0113 (18)
C19	0.046 (3)	0.054 (3)	0.069 (4)	0.004 (3)	0.005 (3)	0.009 (3)
C20	0.041 (3)	0.041 (3)	0.054 (3)	0.003 (2)	−0.001 (2)	0.001 (2)
C21	0.050 (3)	0.063 (3)	0.057 (3)	−0.002 (3)	0.011 (3)	0.005 (3)
C22	0.047 (3)	0.057 (3)	0.091 (5)	−0.008 (3)	0.006 (3)	0.001 (3)
C23	0.045 (3)	0.044 (3)	0.073 (4)	0.002 (2)	−0.003 (3)	−0.001 (3)
C24	0.054 (4)	0.065 (4)	0.104 (5)	−0.018 (3)	0.007 (4)	−0.012 (4)
C25	0.068 (5)	0.081 (4)	0.108 (6)	−0.025 (4)	−0.003 (4)	−0.033 (4)
C26	0.072 (4)	0.084 (4)	0.084 (5)	−0.015 (4)	0.000 (4)	−0.028 (4)
C27	0.048 (3)	0.065 (3)	0.070 (4)	−0.011 (3)	−0.003 (3)	−0.014 (3)
C28	0.041 (3)	0.043 (3)	0.061 (3)	0.000 (2)	−0.005 (2)	−0.002 (2)
C29	0.035 (3)	0.037 (2)	0.057 (3)	0.003 (2)	−0.002 (2)	0.003 (2)
C30	0.042 (3)	0.035 (2)	0.045 (2)	0.005 (2)	−0.006 (2)	−0.0049 (19)
C31	0.043 (3)	0.047 (2)	0.043 (3)	−0.001 (2)	0.007 (2)	0.000 (2)
C32	0.043 (3)	0.043 (3)	0.047 (3)	−0.003 (2)	−0.001 (2)	0.006 (2)
C33	0.072 (4)	0.065 (4)	0.051 (3)	−0.013 (3)	−0.018 (3)	0.007 (3)
C34	0.147 (7)	0.124 (6)	0.086 (5)	0.000 (6)	−0.034 (5)	0.026 (4)
C35	0.096 (5)	0.057 (3)	0.106 (5)	0.001 (4)	−0.033 (4)	0.017 (3)
N6	0.127 (5)	0.101 (4)	0.090 (4)	−0.023 (4)	0.026 (4)	0.006 (3)
C37	0.035 (3)	0.049 (3)	0.040 (3)	−0.013 (2)	0.003 (2)	−0.014 (2)
C38	0.043 (3)	0.060 (3)	0.071 (4)	−0.006 (3)	0.004 (3)	−0.006 (3)
N1	0.039 (2)	0.042 (2)	0.045 (2)	0.0053 (19)	0.0007 (19)	0.0027 (16)
N2	0.041 (2)	0.053 (2)	0.048 (2)	0.008 (2)	0.000 (2)	0.0066 (17)
C17	0.089 (5)	0.078 (4)	0.035 (3)	0.002 (4)	−0.001 (3)	0.021 (3)
N4	0.037 (2)	0.0382 (19)	0.052 (2)	0.0037 (19)	0.0017 (19)	0.0009 (17)
N5	0.048 (3)	0.046 (2)	0.050 (2)	−0.007 (2)	0.009 (2)	−0.0032 (18)
C36	0.111 (6)	0.106 (5)	0.041 (3)	0.004 (5)	−0.008 (4)	−0.021 (3)
O1	0.051 (2)	0.064 (2)	0.0435 (19)	0.0131 (18)	0.0023 (16)	0.0064 (16)
O2	0.042 (2)	0.059 (2)	0.050 (2)	0.0077 (17)	0.0079 (16)	0.0131 (16)
O3	0.038 (2)	0.077 (2)	0.072 (3)	0.009 (2)	0.0085 (17)	0.026 (2)
O4	0.053 (2)	0.057 (2)	0.053 (2)	−0.0091 (18)	0.0068 (17)	−0.0074 (17)
O5	0.042 (2)	0.071 (2)	0.047 (2)	−0.0090 (18)	−0.0001 (16)	−0.0069 (17)
O6	0.040 (2)	0.082 (2)	0.055 (2)	−0.0127 (19)	0.0029 (17)	0.0037 (18)

### Geometric parameters (Å, °)

**Table:**

Ni1—O1	1.805 (3)	C18—H18	0.9300
Ni1—N1	1.833 (3)	C19—N2	1.325 (6)
Ni1—O2	1.852 (3)	C19—H19	0.9300
Ni1—N2	1.900 (3)	C20—O4	1.313 (5)
Ni2—O4	1.816 (3)	C20—C29	1.406 (6)
Ni2—N4	1.832 (4)	C20—C21	1.428 (7)
Ni2—O5	1.857 (3)	C21—C22	1.349 (7)
Ni2—N5	1.894 (4)	C21—H21	0.9300
C1—O1	1.311 (5)	C22—C23	1.415 (7)
C1—C10	1.410 (6)	C22—H22	0.9300
C1—C2	1.417 (7)	C23—C24	1.417 (7)
C2—C3	1.347 (7)	C23—C28	1.425 (7)
C2—H2	0.9300	C24—C25	1.343 (8)
C3—C4	1.413 (7)	C24—H24	0.9300
C3—H3	0.9300	C25—C26	1.380 (8)
C4—C9	1.408 (7)	C25—H25	0.9300
C4—C5	1.420 (7)	C26—C27	1.368 (7)
C5—C6	1.384 (8)	C26—H26	0.9300
C5—H5	0.9300	C27—C28	1.400 (7)
C6—C7	1.377 (8)	C27—H27	0.9300
C6—H6	0.9300	C28—C29	1.449 (6)
C7—C8	1.374 (7)	C29—C30	1.421 (6)
C7—H7	0.9300	C30—N4	1.292 (5)
C8—C9	1.412 (7)	C30—H30	0.9300
C8—H8	0.9300	C31—N4	1.477 (6)
C9—C10	1.446 (6)	C31—C32	1.522 (7)
C10—C11	1.415 (6)	C31—C33	1.543 (7)
C11—N1	1.294 (5)	C31—H31	0.9800
C11—H11	0.9300	C32—O6	1.230 (6)
C12—N1	1.484 (6)	C32—O5	1.288 (5)
C12—C13	1.496 (7)	C33—C34	1.505 (8)
C12—C14	1.540 (7)	C33—C35	1.517 (8)
C12—H12	0.9800	C33—H33	0.9800
C13—O3	1.222 (6)	C34—H34A	0.9600
C13—O2	1.300 (5)	C34—H34B	0.9600
C14—C15	1.519 (7)	C34—H34C	0.9600
C14—C16	1.521 (7)	C35—H35A	0.9600
C14—H14	0.9800	C35—H35B	0.9600
C15—H15A	0.9600	C35—H35C	0.9600
C15—H15B	0.9600	N6—C36	1.372 (7)
C15—H15C	0.9600	N6—N5	1.372 (6)
C16—H16A	0.9600	N6—H6A	0.8600
C16—H16B	0.9600	C37—C36	1.304 (8)
C16—H16C	0.9600	C37—C38	1.307 (6)
N3—C17	1.359 (7)	C37—H37	0.9300
N3—N2	1.393 (6)	C38—N5	1.312 (6)
N3—H3A	0.8600	C38—H38	0.9300
C18—C19	1.312 (6)	C17—H17	0.9300
C18—C17	1.334 (7)	C36—H36	0.9300
			
O1—Ni1—N1	94.55 (15)	C20—C21—H21	119.6
O1—Ni1—O2	176.92 (16)	C21—C22—C23	122.3 (5)
N1—Ni1—O2	86.20 (15)	C21—C22—H22	118.8
O1—Ni1—N2	89.98 (16)	C23—C22—H22	118.8
N1—Ni1—N2	173.95 (16)	C22—C23—C24	122.0 (5)
O2—Ni1—N2	89.49 (16)	C22—C23—C28	118.9 (5)
O4—Ni2—N4	95.23 (16)	C24—C23—C28	119.0 (5)
O4—Ni2—O5	174.97 (15)	C25—C24—C23	122.1 (6)
N4—Ni2—O5	86.79 (16)	C25—C24—H24	119.0
O4—Ni2—N5	89.59 (16)	C23—C24—H24	119.0
N4—Ni2—N5	174.72 (17)	C24—C25—C26	119.2 (6)
O5—Ni2—N5	88.58 (16)	C24—C25—H25	120.4
O1—C1—C10	124.9 (4)	C26—C25—H25	120.4
O1—C1—C2	116.3 (4)	C27—C26—C25	120.9 (6)
C10—C1—C2	118.8 (4)	C27—C26—H26	119.5
C3—C2—C1	121.7 (5)	C25—C26—H26	119.5
C3—C2—H2	119.1	C26—C27—C28	122.2 (5)
C1—C2—H2	119.1	C26—C27—H27	118.9
C2—C3—C4	121.2 (5)	C28—C27—H27	118.9
C2—C3—H3	119.4	C27—C28—C23	116.6 (5)
C4—C3—H3	119.4	C27—C28—C29	124.7 (5)
C9—C4—C3	119.5 (5)	C23—C28—C29	118.6 (5)
C9—C4—C5	119.7 (5)	C20—C29—C30	120.1 (4)
C3—C4—C5	120.7 (5)	C20—C29—C28	120.1 (4)
C6—C5—C4	120.8 (5)	C30—C29—C28	119.8 (4)
C6—C5—H5	119.6	N4—C30—C29	125.7 (4)
C4—C5—H5	119.6	N4—C30—H30	117.1
C7—C6—C5	119.6 (5)	C29—C30—H30	117.1
C7—C6—H6	120.2	N4—C31—C32	106.1 (4)
C5—C6—H6	120.2	N4—C31—C33	115.0 (4)
C8—C7—C6	120.4 (5)	C32—C31—C33	109.4 (4)
C8—C7—H7	119.8	N4—C31—H31	108.7
C6—C7—H7	119.8	C32—C31—H31	108.7
C7—C8—C9	122.4 (5)	C33—C31—H31	108.7
C7—C8—H8	118.8	O6—C32—O5	123.0 (4)
C9—C8—H8	118.8	O6—C32—C31	121.3 (4)
C4—C9—C8	117.1 (5)	O5—C32—C31	115.7 (4)
C4—C9—C10	119.1 (4)	C34—C33—C35	115.7 (6)
C8—C9—C10	123.8 (4)	C34—C33—C31	111.9 (5)
C1—C10—C11	119.6 (4)	C35—C33—C31	111.9 (4)
C1—C10—C9	119.4 (4)	C34—C33—H33	105.5
C11—C10—C9	120.9 (4)	C35—C33—H33	105.5
N1—C11—C10	125.5 (4)	C31—C33—H33	105.5
N1—C11—H11	117.3	C33—C34—H34A	109.5
C10—C11—H11	117.3	C33—C34—H34B	109.5
N1—C12—C13	107.0 (4)	H34A—C34—H34B	109.5
N1—C12—C14	111.9 (4)	C33—C34—H34C	109.5
C13—C12—C14	112.3 (4)	H34A—C34—H34C	109.5
N1—C12—H12	108.5	H34B—C34—H34C	109.5
C13—C12—H12	108.5	C33—C35—H35A	109.5
C14—C12—H12	108.5	C33—C35—H35B	109.5
O3—C13—O2	122.7 (5)	H35A—C35—H35B	109.5
O3—C13—C12	122.3 (4)	C33—C35—H35C	109.5
O2—C13—C12	115.0 (4)	H35A—C35—H35C	109.5
C15—C14—C16	112.8 (5)	H35B—C35—H35C	109.5
C15—C14—C12	112.4 (4)	C36—N6—N5	107.1 (5)
C16—C14—C12	111.5 (5)	C36—N6—H6A	126.5
C15—C14—H14	106.5	N5—N6—H6A	126.5
C16—C14—H14	106.5	C36—C37—C38	106.9 (5)
C12—C14—H14	106.5	C36—C37—H37	126.5
C14—C15—H15A	109.5	C38—C37—H37	126.5
C14—C15—H15B	109.5	C37—C38—N5	113.3 (5)
H15A—C15—H15B	109.5	C37—C38—H38	123.3
C14—C15—H15C	109.5	N5—C38—H38	123.3
H15A—C15—H15C	109.5	C11—N1—C12	120.3 (4)
H15B—C15—H15C	109.5	C11—N1—Ni1	126.8 (3)
C14—C16—H16A	109.5	C12—N1—Ni1	112.9 (3)
C14—C16—H16B	109.5	C19—N2—N3	103.4 (4)
H16A—C16—H16B	109.5	C19—N2—Ni1	127.7 (3)
C14—C16—H16C	109.5	N3—N2—Ni1	128.1 (4)
H16A—C16—H16C	109.5	C18—C17—N3	106.5 (5)
H16B—C16—H16C	109.5	C18—C17—H17	126.7
C17—N3—N2	109.1 (5)	N3—C17—H17	126.7
C17—N3—H3A	125.4	C30—N4—C31	120.0 (4)
N2—N3—H3A	125.4	C30—N4—Ni2	126.3 (3)
C19—C18—C17	108.3 (5)	C31—N4—Ni2	113.5 (3)
C19—C18—H18	125.8	C38—N5—N6	104.2 (4)
C17—C18—H18	125.8	C38—N5—Ni2	125.6 (4)
C18—C19—N2	112.6 (5)	N6—N5—Ni2	130.0 (3)
C18—C19—H19	123.7	C37—C36—N6	108.4 (5)
N2—C19—H19	123.7	C37—C36—H36	125.8
O4—C20—C29	125.0 (4)	N6—C36—H36	125.8
O4—C20—C21	115.9 (4)	C1—O1—Ni1	127.4 (3)
C29—C20—C21	119.1 (4)	C13—O2—Ni1	116.1 (3)
C22—C21—C20	120.9 (5)	C20—O4—Ni2	126.8 (3)
C22—C21—H21	119.6	C32—O5—Ni2	115.1 (3)

### Hydrogen-bond geometry (Å, °)

**Table:**

*D*—H···*A*	*D*—H	H···*A*	*D*···*A*	*D*—H···*A*
C7—H7···O2^i^	0.93	2.59	3.476 (6)	159.
C14—H14···O6^ii^	0.98	2.42	3.318 (6)	153.
C18—H18···O6^iii^	0.93	1.87	2.798 (5)	178.
C37—H37···O3^iv^	0.93	1.85	2.756 (5)	163.

Symmetry codes: (i) −*x*+3/2, −*y*, *z*+1/2; (ii) *x*, *y*−1, *z*; (iii) −*x*+3/2, −*y*+1, *z*−1/2; (iv) −*x*+3/2, −*y*+1, *z*+1/2.

### Table 3 Weak Cg-Cg intermolecular interactions of (I) (Å)

**Table:**

distance	Cg1—Cg10	Cg3—Cg9	Cg4—Cg7	Cg4—Cg9
centroid-centroid distance	3.940 (3)	3.709 (2)	3.526 (3)	3.932 (3)

Notes: CgI—CgJ = centroid-centroid distance between Plane I and J (Å); Cg1: Ni1/O2/C13/C12/N1; Cg3: Ni1/O1/C1/C10/C11/N1; Cg4: C1/C2/C3/C4/C9/C10; Cg7: Ni2/O5/C32/C31/N4; Cg9: Ni2/O4/C20/C29/C30/N4; Cg10: C20/C21/C22/C23/C28/C29.
